# The soluble neurexin-1β ectodomain causes calcium influx and augments dendritic outgrowth and synaptic transmission

**DOI:** 10.1038/s41598-020-75047-z

**Published:** 2020-10-22

**Authors:** Keimpe D. B. Wierda, Trine L. Toft-Bertelsen, Casper R. Gøtzsche, Ellis Pedersen, Irina Korshunova, Janne Nielsen, Marie Louise Bang, Andreas B. Kønig, Sylwia Owczarek, Michelle D. Gjørlund, Melanie Schupp, Elisabeth Bock, Jakob B. Sørensen

**Affiliations:** 1 Neurosecretion Group, Department of Neuroscience, Faculty of Health and Medical Sciences, University of Copenhagen, Blegdamsvej 3B, 2200 Copenhagen N, Denmark; 2 Laboratory of Neural Plasticity, Department of Neuroscience, Faculty of Health and Medical Sciences, University of Copenhagen, Blegdamsvej 3B, 2200 Copenhagen N, Denmark

**Keywords:** Synaptic vesicle exocytosis, Cellular neuroscience, Molecular neuroscience

## Abstract

Classically, neurexins are thought to mediate synaptic connections through *trans* interactions with a number of different postsynaptic partners. Neurexins are cleaved by metalloproteases in an activity-dependent manner, releasing the soluble extracellular domain. Here, we report that in both immature (before synaptogenesis) and mature (after synaptogenesis) hippocampal neurons, the soluble neurexin-1β ectodomain triggers acute Ca^2+^-influx at the dendritic/postsynaptic side. In both cases, neuroligin-1 expression was required. In immature neurons, calcium influx required N-type calcium channels and stimulated dendritic outgrowth and neuronal survival. In mature glutamatergic neurons the neurexin-1β ectodomain stimulated calcium influx through NMDA-receptors, which increased presynaptic release probability. In contrast, prolonged exposure to the ectodomain led to inhibition of synaptic transmission. This secondary inhibition was activity- and neuroligin-1 dependent and caused by a reduction in the readily-releasable pool of vesicles. A synthetic peptide modeled after the neurexin-1β:neuroligin-1 interaction site reproduced the cellular effects of the neurexin-1β ectodomain. Collectively, our findings demonstrate that the soluble neurexin ectodomain stimulates growth of neurons and exerts acute and chronic effects on *trans*-synaptic signaling involved in setting synaptic strength.

## Introduction

A great diversity in properties of synapses in the central nervous system is determined by a variety of synaptic cell adhesion molecules (CAMs), which play a key role in specifying and tuning synaptic plasticity^1^. Neurexins (NXs) constitute a family of CAMs, which encompasses three members, NX1-3, expressed as long (α-NXs) and short (β-NXs) isoforms driven by alternative promotors^2–4^ yielding thousands of isoforms in the brain^5,6^. Neurexins interact with key synaptic organizers to initially orchestrate discrete synaptic signaling pathways^1,7^, and a number of ligand proteins including postsynaptically expressed neuroligins (NLs) and Leucine-Rich Repeat Trans Membrane proteins (LRRTMs) to mediate synapse formation, maturation and function^1,2,8–15^. Other β-NX interaction partners are presynaptic CIRL1/Latrophilin-1^16^, secreted synaptic protein cerebellin^17^, neuroxophilin^18^, dystroglycan^18^, as well as GABA_A_^19^ and acetylcholine receptors^20^.

Heterologous overexpression of NLs or NXs in non-neuronal cells promotes initial synapse formation in co-cultured neurons^21,22^ through NX:NL-pairing^23^. After synapse formation the NX:NL interaction is required for synapse stabilization and maturation^24,25^. Furthermore, the NX:NL *trans* synaptic connection modulates presynaptic release properties via retrograde signaling^26^. For instance, overexpression of NL-1 in immature neurons increases the recycling vesicle pool size^27^ and postsynaptic expression of NL-1 in brain slices increases the presynaptic release probability^26^. Conversely, in *Caenorhabditis elegans* neuromuscular junction, the NX:NL interaction mediates a retrograde signal, which inhibits fusion of synaptic vesicles distal to Ca^2+^ entry sites^28^ mediated by NX binding to N-type voltage-gated calcium channels (VGCC)^29^.

Endogenous NLs grant properties onto their resident synapses^30^. Mice with genetic deletion of NL-1 have a reduced NMDA/AMPA-ratio, which is typical for juvenile synapses^30,31^. In contrast, NL-1 overexpression causes increases in AMPA EPSC amplitude and NMDA/AMPA-ratio^30^. These changes depend on neuronal activity, indicating a role in synapse validation and function^27,30^. Conditional knockout of endogenous β-NXs in mice markedly impairs neurotransmitter release and β-NXs are similarly involved in synaptic regulation processes and control synaptic strength specifically via postsynaptic synthesis of endocannabinoids, which exert their function presynaptically through the CB1 receptor^32^. Taken together, the NX:NL *trans* synaptic connection has the potential to dictate bidirectional changes in (pre-)synaptic strength. A still open question is whether NXs have the potential for not only dynamic tuning, but also acutely regulating synaptic transmission. An acute role in regulating synapse function might be anticipated from the observation that NXs—like NLs—are sequentially cleaved by α- and γ-secretases herewith shedding the ectodomain and thus releasing the intracellular C-terminal fragment^33–36^. It is well-established that ectodomain shedding of several neural CAMs, including *N*-cadherin, NCAM and NL-1, can modulate the adhesive, synaptogenic and signaling properties of these CAMs^35,37–42^. However, for the soluble NX-1β ectodomain the physiological role in neuronal growth and acute regulation of synaptic transmission is not clear.

Here, we studied the functional ramifications of the NX-1β ectodomain and a synthetic peptide modeled on the minimal binding sequence in NX-1β for NL-1. Notably, we find that both the ectodomain and the peptide potently stimulate neuritogenesis in immature neurons and induce an acute (within a few seconds) increase in synaptic strength in mature glutamatergic neurons, which progresses into an activity-dependent homeostatic down-regulation within two hours. These effects are found to be NL-1 and Ca^2+^-dependent, and indicate that the NX-1β ectodomain is potentially involved in acutely adjusting synaptic strength.

## Results

### NX-1β ectodomain induces Ca^2+^ influx and neurite outgrowth in immature neurons in a NL-1-dependent manner

To investigate the potential function of the ectodomain of NX-1β, we used a NX-1β ectodomain lacking the splice site 4 (SS4), synthesized as a Fc-chimera (henceforth referred to as NX-1βe). It is well-known that NX-1β lacking SS4 interacts with NL-1^9,43,44^. Consistently, surface plasmon resonance analysis confirmed binding of NX-1βe to recombinant NL-1 (Fig. S1a). The dissociation constant (K_D_) was 32 nM, (Fig. S1a) however the covalent immobilization of the receptor, i.e., NL-1, to the chip at least partially masks the binding interface, and does not mimick native NL-1 in the cell surface environment. Thus, a lower concentration of the protein might yield a functional response.

**Figure 1:**
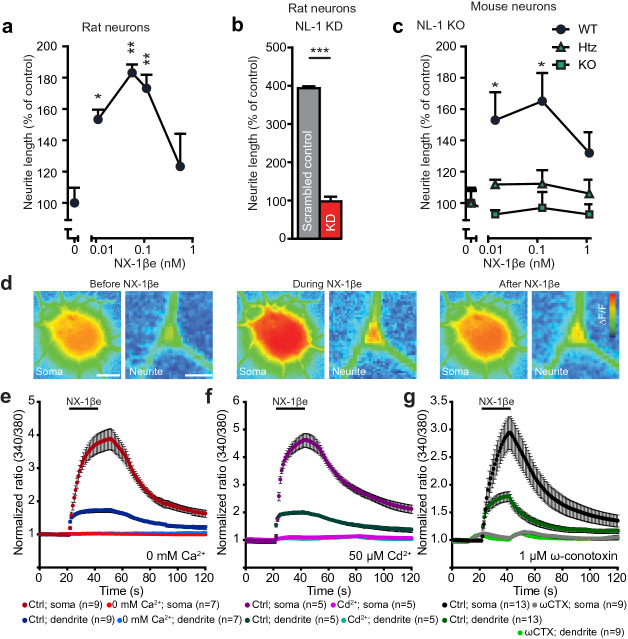
A soluble NX-β ectodomain (NX-1βe) induces neurite outgrowth and Ca^2+^ influx. (**a**–**c**) Effects of NX-1βe on neurite outgrowth in cultures of rat (**a**,**b**) and mouse (**c**) hippocampal neurons 24 h after application. The neuritogenic effect of NX-1βe was absent in rat neurons after knock down of NL-1 (KD; cells transfected with the shRNA containing p-GFP-V-RS vector were identified by their green fluorescence), or in NL-1 knockout (KO) mouse neurons, compared to wildtype (*WT* wildtype, *Htz* heterozygous; *n* = 4–7 cultures). (**d**) Fluorescence micrographs of an immature neuron (DIV 1) soma and a piece of neurite from the same cell before NX-1βe application (left), during (middle), and after wash-out (right). The cell was loaded with Fura-2AM and shown is the ratio of fluorescence after 340/380 nm excitation, which reports on [Ca^2+^]_i_. Scale bar = 3 µm (soma), 1 µm (neurite). (**e**–**g**) Quantification of 340/380 fluorescence ratios in soma and neurites before, during, and after 20 s exposure to NX-1βe shows a reversible increase in cytoplasmic [Ca^2+^]. (**e**) The omission of Ca^2+^ from the solution (0 mM Ca^2+^) abrogated the [Ca^2+^]_i_ increase, which was also seen upon addition of the Cd^2+^ (**f**). (**g**) The N-type Ca^2+^ channel blocker ω-conotoxin blocked most Ca^2+^-influx.

Initially, we studied the effect of NX-1βe on neurite outgrowth in immature cultured hippocampal neurons (DIV 1). Primary cultures of neurons are known to express both NX-1 and NL-1 prior to synapse formation^45^. Application of NX-1βe strongly stimulated neurite outgrowth within 24 h after seeding, in a concentration-dependent manner. The resultant bell-shaped dose-dependency with the most efficacious concentrations was found between 0.01 and 0.1 nM (rat neurons: Fig. 1a; mouse neurons: Supplementary Fig. S1b). At higher concentrations, no effect was seen. Importantly, the neuritogenic effect of NX-1βe was abrogated by shRNA-induced knockdown of NL-1 in rat hippocampal neurons (Fig. 1b). Note that for shRNA-experiments neurons were plated on L929 fibroblasts and given a more nutrient-rich medium; possibly for this reason, the neurite length was larger in this experiment (Fig. 1a,b). Similarly, in primary neurons isolated from NL-1 knockout (KO) mice the effect of NX-1βe was absent (Fig. 1c). This specifies that the role of NX-1βe in neurite outgrowth depends on NL-1 expression.

Calcium is required for neuronal development^46^, and α-NXs have been implicated in the regulation and targeting of Ca^2+^ channels^24^. The neuritogenic effect of the NX-1βe might therefore require intracellular Ca^2+^ signaling. Indeed, in hippocampal cultures ω-conotoxin MVIIA (an inhibitor of *N*-type VGCCs) reduced the neuritogenic effects of NX-1βe (Supplementary Fig. S1c). To test whether NX-1βe itself modifies Ca^2+^ homeostasis, we performed experiments in immature neurons loaded with the membrane permeable fluorescent Ca^2+^-sensitive dye Fura-2AM. Strikingly, acute application of NX-1βe (~ 55 pM, i.e. the concentration leading to maximal outgrowth, Fig. 1a) led to a strong increase in the intracellular Ca^2+^-concentration ([Ca^2+^]_i_) in both neurites and the somatic compartment (Fig. 1d,e). The increase was mediated by Ca^2+^-influx since omitting Ca^2+^ from the bath abrogated the effect (Fig. 1e). Furthermore, it was blocked by cadmium ions (Fig. 1f), and ω-conotoxin, (Fig. 1g), indicating the involvement of *N*-type VGCCs.

Several neural CAMs, including NCAM and CIRL1 are known to function as survival factors^47,48^. We tested whether NX-1βe possessed protective properties in hippocampal and cerebellar granule neurons by H_2_O_2_-induced cell death and potassium deprivation-induced apoptosis. We found that NX-1βe promoted survival of both hippocampal and cerebellar granule neurons, respectively (Supplementary Fig. S1d). As a control, treatment with previously identified neuroprotective compounds, S100A4 protein^49^ and insulin-like growth factor-1^50^ was used and found to rescue both cell types (Supplementary Fig. S1f,g). Thus the NX-1β ectodomain functions, in accordance with other neural CAMs, as a survival factor.

Collectively, these data show that the NX-1βe exerts potent effects at low concentrations (Fig. 1a, Supplementary Fig. S1b,d,e) on neuronal survival and neuritogenesis in vitro, in a process that depends obligatorily on the expression of NL-1. Possibly, NL-1 acts as an activation receptor for NX-1βe, which upon binding causes Ca^2+^-influx utilizing another part of the ectodomain. Alternatively, NX-1βe induced Ca^2+^-influx and induction of neurite outgrowth might depend on NX-1βe:NL-1 binding itself, in which case only the binding interface of the NX-1β ectodomain would be needed.

### A synthetic NX-1β-derived peptide, Neurexide, mimics the effect of NX-1β ectodomain

**Figure 2:**
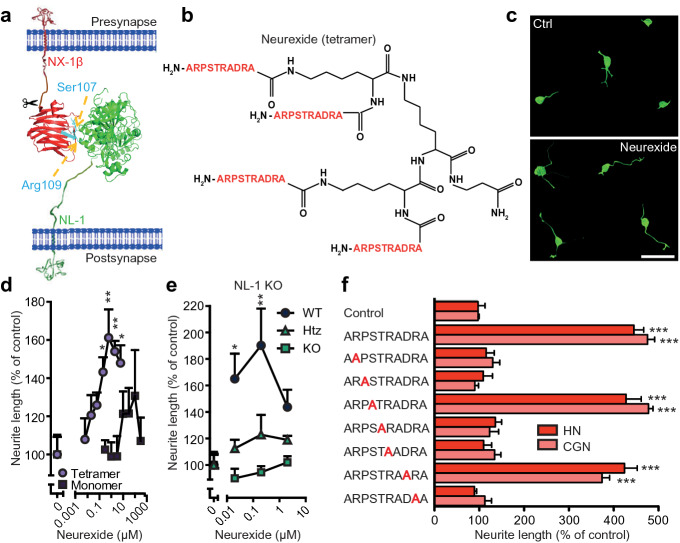
Neurexide, a peptide modeled after the NX-1β binding site for NL-1, also stimulates neurite outgrowth. (**a**) Ribbon rendition of the NX-1β (red):NL-1 (green) complex, showing the localization of Neurexide (blue) (PDB ID: 3BIW), modified from^72^. Orange arrows show localization of residues in NX-1β involved in interaction with NL-1. An arbitrarily positioned scissor signifies shedding of the NX-1βe. (**b**) Schematic presentation of the tetrameric version of Neurexide peptide structure. (**c**) Representative images of control or 0.6 µM tetrameric Neurexide treated DIV1 cultures, scale bar = 20 µm. (**d**,**e**) Effects on neurite outgrowth (DIV1) in cultures of rat (**d**) and mouse (**e**) hippocampal neurons 24 h after application of Neurexide (n = 4 cultures). The monomeric versus tetrameric peptide was tested in rat hippocampal neurons. (**e**) The effect of Neurexide on neurite formation was absent in NL-1 knockout (KO) neurons, compared to wildtype (*WT* wildtype, *Htz* heterozygous). (**f**) Sequence-specificity of tetrameric Neurexide (DIV1, n = 4 cultures). Single substitutions of Arg2, Pro3, Thr5, Arg6, or Arg9 with alanine abrogated the effect of Neurexide on neurite formation. *p < 0.05, **p < 0.01, ***p < 0.001.

To distinguish between these possibilities we next investigated whether a minimal peptide modeled after the NX-1β:NL-1 interface could mimic the effect of NX-1βe. Based on the crystal structures of the NX-1β:NL-1 complex^43,51,52^ we designed a 10-mer peptide, termed Neurexide (*Neurex*in pept*ide*; sequence in single-letter code: ARPSTRADRA), modeled after the NX-1β binding site for NL-1 (Fig. 2a,b). The peptide was synthesized either as a C-terminally amidated monomer or a multimer (dimer and tetramer) on a lysine backbone (Fig. 2b and Supplementary Fig. S2a). Similar to NX-1βe, the tetrameric form of Neurexide strongly induced neurite outgrowth in rat or mouse primary hippocampal neurons, whereas the monomeric form was much less effective. The dimeric peptide induced an intermediate effect at higher concentrations (Fig. 2c,d and Supplementary Fig. S2b,c). The tetrameric form was therefore used in subsequent experiments. As for the NX-1βe, the Neurexide-induced effect on neurite outgrowth was abolished by knockdown (Supplementary Fig. S2d) or KO (Fig. 2e) of NL-1. To verify the specificity of the Neurexide-induced neuritogenic response Neurexide-derived peptides with scrambled or reversed sequences or single alanine substitutions of Arg2, Pro3, Thr5, Arg6, or Arg9 was employed. None showed activity (Fig. 2f and Supplementary Fig. S2e). Neurexide predominantly increased dendritic growth, as evident after immunostaining against a dendritic marker (MAP2), while axonal growth was unaffected (marked with Neurofilament) (Supplementary Fig. S3).

In the neuronal survival assay, Neurexide displayed a significant survival effect on hippocampal neurons, whereas a noticeable (but statistically insignificant) trend in cerebellar granule neurons survival was seen (Supplementary Fig. S1e). As for NX-1βe, the stimulation of outgrowth by Neurexide in hippocampal neurons was inhibited by ω-conotoxin MVIIA (Supplementary Fig. S2f), indicating that Neurexide promotes outgrowth in a Ca^2+^-dependent manner similar to that of NX-1βe. We finally tested whether Neurexide interacts with NL-1. Surface plasmon resonance analysis indeed showed that this interaction occurs with sub-μM affinity (Supplementary Fig. S2g, estimated K_D_ = 480 nM). The lower affinity compared to full-length NX-1βe correlates with the higher concentration of Neurexide needed for optimal effect (Fig. 2d).

In sum, the effect of the NX-1β ectodomain on neurite outgrowth and survival is mimicked by a minimal peptide modeled after the NX-1β:NL1 binding site, indicating that we have identified the minimal region of the ectodomain required for this effect.

### The NX-1β ectodomain acutely enhances glutamatergic synaptic transmission

After having established that both the NX-1β ectodomain and the synthetic peptide Neurexide affect neuritogenesis, we set out to investigate whether the compounds affect synaptic transmission in already formed synapses. To this end, we used rat hippocampal glutamatergic neurons grown in autaptic culture for 10–14 days^53^. Since only one autoinnervating neuron is present per glial island, differences in neuronal survival will not affect the results in this preparation. In voltage clamp experiments individual action potentials (APs) are evoked upon brief depolarization, while spontaneous APs are effectively prevented when keeping the membrane potential at -70 mV during inter-stimulus intervals. Therefore, it is possible to assess both evoked postsynaptic currents and mini events in the same experiment without the use of tetrodotoxin (TTX). After establishing a whole-cell configuration, the neurons were stimulated by AP pairs (inter-stimulus interval 50 ms) every 20 s. First evoked EPSC (eEPSC) amplitude and Paired-Pulse Ratio (PPR) were quantified for each stimulus pair, and miniature EPSCs (mEPSCs) were analyzed in-between stimuli.

**Figure 3:**
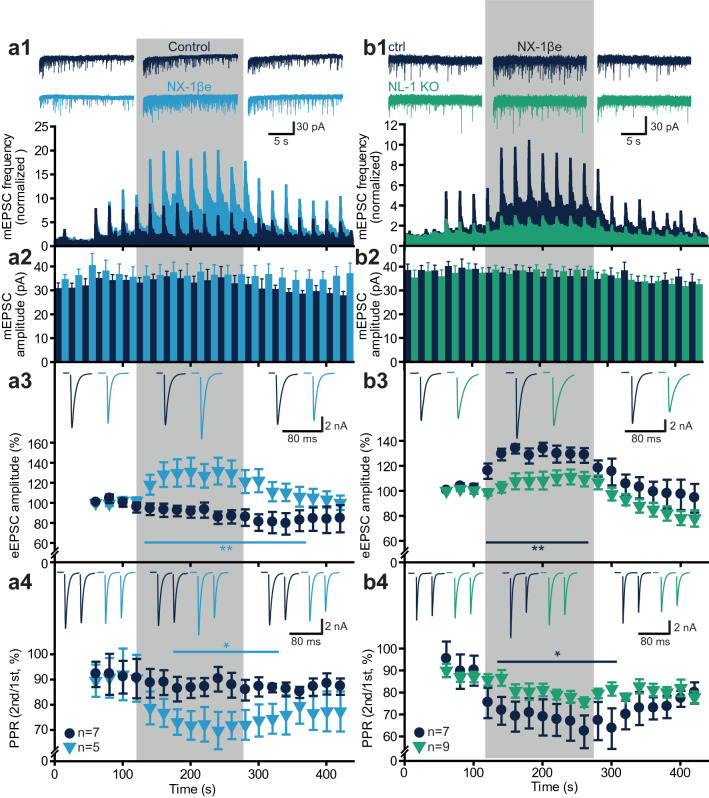
Acute exposure to NX-1βe stimulates glutamatergic synaptic transmission in the presence of NL-1. (**a1**–**3**) Autaptic glutamatergic neurons were patch-clamped in whole cell voltage-clamp configuration and stimulated by action potential pairs (inter stimulus interval 50 ms) every 20 s. mEPSC frequency (**a1**) was quantified between stimuli, together with mEPSC amplitude (**a2**) eEPSC amplitude (**a3**) and Paired-Pulse Ratio (PPR) (**a4**) for each paired stimulation. NX-1βe was applied in the bath (shaded area); ‘Control’ denotes superfusion with a control solution without NX-1βe. NX-1βe reversibly increased the mEPSC frequency, and the eEPSC amplitude, whereas the PPR was reduced, indicating increased release probability. Note the post-stimulation induced (Ca^2+^-dependent) periodic bursts in mEPSC release frequency. (**b1**–**4**) Application of NX-1βe to neurons isolated from NL-1 KO mice (NL-1 KO) and wildtype littermates (ctrl). In the absence of NL-1, NX-1βe was ineffective in increasing mEPSC frequency (**b1**), eEPSC amplitude (**b3**) and decreasing paired pulse ratio (PPR, **b4**). This is consistent with NL-1 constituting the main target of NX-1βe.

We acutely exposed rat glutamatergic neurons to NX-1βe using a local superfusion multibarrel system during the recording (concentration ~ 55 pM, similar to the concentration causing maximal stimulation of neuritogenesis, Fig. 1a). Strikingly, local application of NX-1βe acutely enhanced synaptic transmission manifested as an increased frequency of mEPSC release (Fig. 3a1) and an increase in eEPSC amplitude (Fig. 3a3). This occurred concomitantly with a decrease in PPR (Fig. 3a4), indicative of an increase in synaptic release probability^54^. The mEPSC amplitude remained unchanged (Fig. 3a2). Importantly, the effects of NX-1βe were reversible upon wash (Fig. 3a1–4, Supplementary Fig. S4), indicating that this type of synaptic enhancement requires continued presence of NX-1βe. The increase in mEPSC frequency and decrease of PPR indicate that the effect of NX-1βe is presynaptic. Note that the mEPSC frequency sharply increases after each paired stimulation due to increases in calcium concentrations, resulting in a sawtooth pattern. During NX-1βe application the activity-induced increase in mEPSC frequency is even more pronounced (higher peaks) and lasts longer (slower relaxation), indicating that NX-1βe affects presynaptic calcium homeostasis. Indeed, when glutamatergic neurons were incubated with BAPTA-AM to chelate Ca^2+^, the vast majority of mEPSCs were blocked, also during NX-1βe application (Supplementary Fig. S4). This shows that mEPSCs are Ca^2+^-dependent both in the presence and absence of NX-1βe.

In line with results above on neuritogenesis, we asked whether the effect of NX-1βe depends on NL-1 expression. Applying NX-1βe to WT mouse neurons resulted in similar changes (Fig. 3b1–3) both on mEPSC frequency, eEPSC amplitude and PPR, which establishes the effect of NX-1βe also in mouse neurons. In contrast, in neurons from NL-1 KO littermates the effects were largely abrogated (Fig. 3b1–4), indicating that the effect mostly depends on NL-1 expression.

Strikingly, application of our peptide Neurexide (10 μM, Supplementary Fig. S5) mimicked the effects of NX-1βe on mEPSC frequency, eEPSC amplitude and PPR, whereas again the mEPSC amplitude was unaffected. Overall, our data show that the NX-1β ectodomain acutely enhances synaptic function in glutamatergic neurons through an increase in release probability. This increase is mimicked by Neurexide emulating the NX1β:NL-1 interaction site and therefore it is not caused by the intracellular part of NX-1β (see “Discussion”). This shows that NX1βe can modify synaptic efficacy on a time scale of seconds in a NL1-dependent manner.

### The NX-1β ectodomain causes Ca^2+^-influx via NMDA-receptors

**Figure 4:**
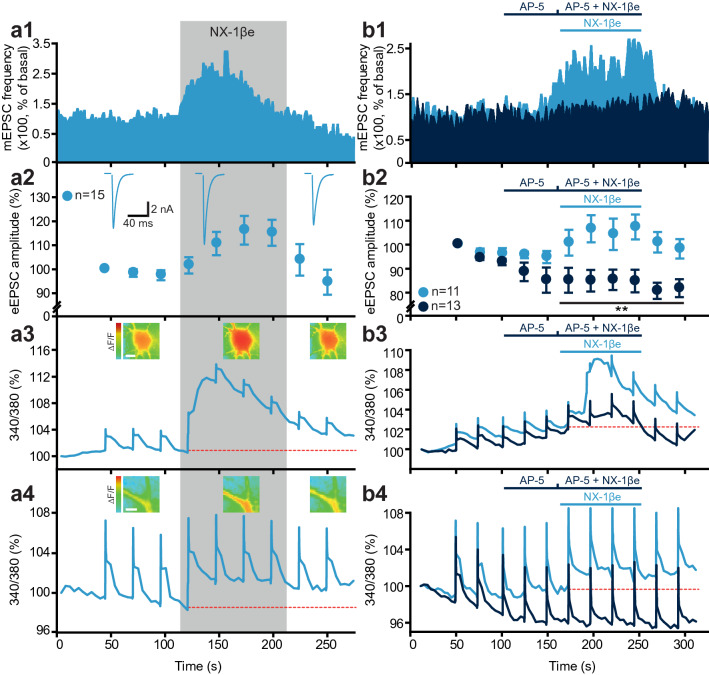
Acute exposure to NX-1βe stimulates postsynaptic Ca^2+^-influx through NMDA-receptors in mature neurons (DIV 10–14). (**a1**–**4**) Autaptic glutamatergic neurons were subjected to patch clamp with (membrane impermeable) Fura-2 in the pipette. Electrophysiological and fluorescence measurements were performed simultaneously. NX-1βe was applied during the grey shaded time period, which led to increased mEPSC frequency (**a1**), increased eEPSC amplitude (**a2**), increased 340/380 fluorescence ratio in cell soma (**a3**), and in dendrites (**a4**), indicative of an increase in cytoplasmic [Ca^2+^]. Note that the increase in fluorescence ratio was much smaller in these cells compared to immature neurons (Fig. 1). (**b1**–**b4**) Blocking NMDA receptors with AP5 during NX-1βe application blocked the increase in mEPSC frequency (**b1**), and eEPSC amplitude (**b2**), as well as the increase in 340/380 fluorescence amplitude in cell soma (**b3**) and dendrites (**b4**). These data demonstrate that Ca^2+^-influx via NMDA-receptors is a prerequisite for upregulating synaptic strength.

To identify the intracellular events, which lead to increased presynaptic release upon exposure to NX-1βe, we loaded autaptic glutamatergic neurons with Fura-2 and combined electrophysiological measurements with fluorescence imaging. Application of NX-1βe rapidly led to an increase in intracellular [Ca^2+^], measured as an increase in 340/380 nm fluorescent ratio in the cell body and in dendrites (Fig. 4a3–4). Note, the increase in mEPSC frequency and eEPSC amplitude were verified in the same cells (Fig. 4a1–2). Previous experiments show that NL-1 interacts with and stabilizes NMDA-receptors at the synapse^30,55^. Therefore, we asked if the source of the Ca^2+^ increase might be influx through NMDA-receptors. Indeed, superfusion with the NMDA-blocker AP-5 abolished both the NX-1βe induced increase in intracellular [Ca^2+^] (Fig. 4b3–4), and the effect of NX-1βe on mEPSC frequency and eEPSC amplitude (Fig. 4b1,b2). Note that AP5 applied alone already reduced Ca^2+^ influx during AP stimulation (Fig. 4b4), indicating that our experimental conditions do allow for NMDA-receptor stimulation. Thus Ca^2+^-influx through NMDA-receptors is a prerequisite for the stimulatory effect of NX-1βe on presynaptic release.

NMDA-receptors are most commonly found postsynaptically, where they interact with NL-1, but they can also be expressed presynaptically^56^. Given the finding that NX-1βe affects presynaptic release and N-type VGCC in developing neurons, we wanted to investigate the effect of NX-1βe on presynaptic [Ca^2+^], which could be due to presynaptic NMDA-receptors^56^ or VGCCs. An effect on Ca^2+^ channels could be expected if the added NX-1βe interferes with presynaptic NMDA-receptors or Ca^2+^ channels^24,57^. Fura-infusion into autaptic neurons does not allow for distinction between pre- and postsynaptic compartments, so in other experiments we expressed a genetically encoded Ca^2+^-sensor fused to synaptophysin, syGCaMP2, which is targeted to synaptic vesicles^58^. Patching the cells and stimulating with short AP trains (10 stimuli @ 40 Hz) before, during and after NX-1βe application revealed clear increases in fluorescence, as expected (Supplementary Fig. S6). We found again NX-1βe increased eEPSC amplitude and concomitantly decreased PPR (Supplementary Fig. S6d,f). However, NX-1βe application did not modify the activity dependent fluorescence increase (ΔF/F), indicating that AP-induced increases in [Ca^2+^]_i_ were unaffected. However, the basal fluorescence slightly increased upon application of NX-1βe, suggesting that resting presynaptic [Ca^2+^] was mildly affected by NX-1βe (Supplementary Fig. S6c). The signal-to-noise relationship of a genetically expressed Ca^2+^ indicator is generally less than that of Fura-2, therefore small changes in presynaptic [Ca^2+^]_i_ might be underestimated. Together, these data support the notion that [Ca^2+^]_i_ is increased by NX-1βe, which leads to a potentiation of synaptic release (see “Discussion”).

### Long-term (hours) exposure to the NX-1β ectodomain homeostatically down regulates the readily-releasable pool of vesicles

Previous investigations making use of the NX-1βe added to neuronal cultures reported a decrease in mEPSC frequency^59^, which correlated with impaired NL-1-dependent synapse formation, presumably due to inhibition of the NX1:NL1 interaction^22,59^. In those experiments, the NX-1β ectodomain was added during the period of active synaptogenesis over longer time (2–3 days) and at higher concentrations than in the present study. The different findings prompted us to investigate whether the effect of the ectodomain might be time or activity dependent. Indeed, after exposure of autaptic neurons to the NX-1βe for 10 days during the period of synaptogenesis (from DIV 1 to 10), the mEPSC frequency and eEPSC amplitudes were severely reduced (Supplementary Fig. S7a,d). In contrast, the mEPSC amplitude and decay time were unchanged (Supplementary Fig. S7b,c), indicating a presynaptic effect. In parallel experiments, NX-1βe was applied for only 2 h to mature cultures to investigate synaptic adaptation independent of synapse development. Intriguingly, 2 h of NX-1βe exposure of mature neurons led to identical changes in synaptic features (DIV 10–14 days; Supplementary Fig. S7), while overnight exposure to NX-1βe did not affect synapse number or neuronal morphology (Supplemental Fig. S10). Taken together, the effects seen here are not due to NX-1βe induced differences in synaptogenesis (see also below).

**Figure 5:**
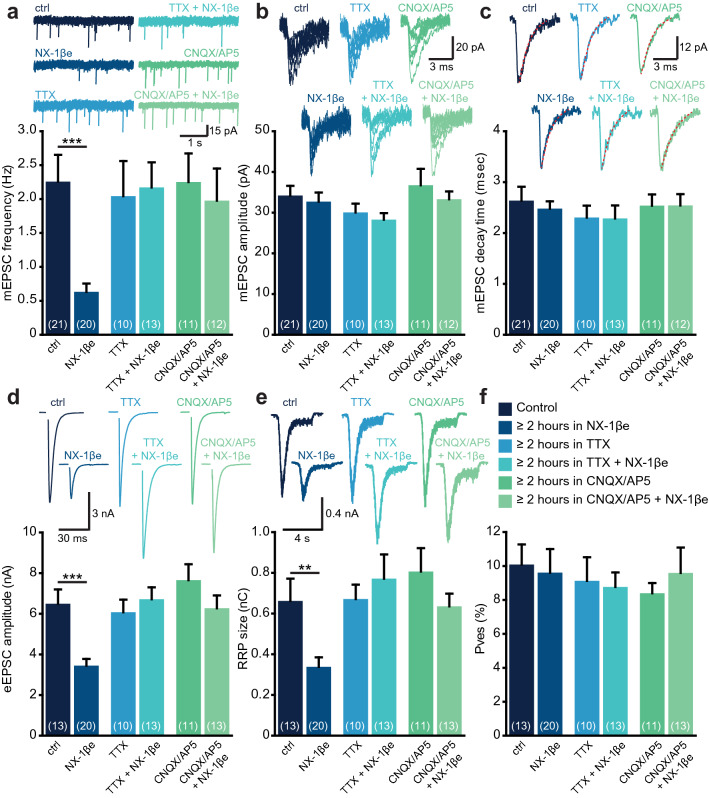
Activity-dependent reduction in the Readily-Releasable Pool (RRP) of vesicles by long-term exposure to NX-1βe. (**a**–**f**) Exposure to NX-1βe for ~ 2 h resulted in a decrease in mEPSC frequency (**a**), a decrease in eEPSC amplitude (**d**), and a decrease in RRP size as estimated by sucrose application (**e**). The mEPSC amplitude (**b**), and decay time (**c**), as well as the vesicular release probability (P_ves_) were unchanged (**f**). When NX-1βe exposure was combined with either Tetrodotoxin (TTX) to block neuronal activity or CNQX/AP5 to block ionotropic glutamatergic neurotransmission, the effect of long-term NX-1βe exposure was abrogated.

The acute upregulation of synaptic strength by NX-1βe identified above might indirectly lead to down-regulation of vesicular release, in a homeostatic plasticity regulative manner which counteracts the global increase in synaptic strength^60,61^. To investigate this point, we added either TTX or a mixture of CNQX and AP5, to block ionotropic glutamate receptors, immediately before adding the NX-1β ectodomain and compared these groups to neurons that were treated only with NX-1βe for 2 h. Also in this independent experimental series 2 h of NX-1βe exposure—when added alone—reduced mEPSC frequency, and eEPSC amplitude (Fig. 5a,d). mEPSC amplitude and decay were unaffected (Fig. 5b,c). Strikingly, application of either TTX or CNQX/AP5 abrogated the effects of NX-1βe (Fig. 5a,d), indicating that neural activity and ionotropic glutamatergic neurotransmission are both prerequisites for long-term NX-1βe induced homeostatic down-regulation of synaptic strength.

Application of hypertonic sucrose solution can be used to probe the readily-releasable pool (RRP) of vesicles^62^. The Rosenmund and Stevens experiments showed that the RRP was depressed in neurons treated with long-term NX-1βe (Fig. 5e), while the vesicular release probability (eEPSC charge divided by the sucrose pool) remained unchanged (Fig. 5f. The same was found in another independent set of experiments (Supplementary Fig. S7e,f; The reduction in RRP was complete within 2 h of exposure to NX-1βe and did not change, even when NX-1βe was present continuously for 10 days (Supplementary Fig. S7e,f). This reduction of RRP size sufficiently explains the inhibition of spontaneous and evoked synaptic transmission caused by long-term NX-1βe exposure.

Overall, these results show that the calcium dependent increase in vesicular release probability induced by acute addition of NX-1βe leads to a secondary long-term activity-dependent down-regulation of the RRP.

### Endogenous metalloproteases and α-secretases regulate the size of the readily-releasable pool

**Figure 6:**
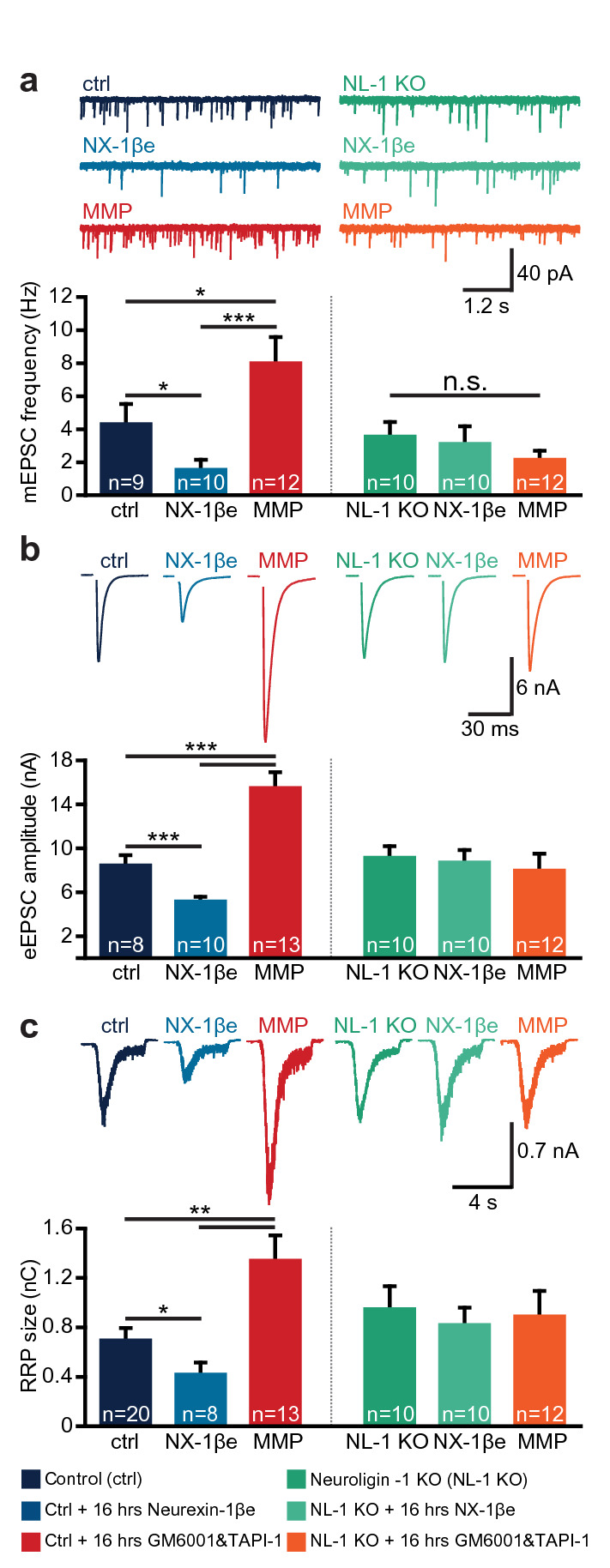
Shedding of endogenous proteins by metalloproteases affects synaptic transmission in neuronal cultures expressing NL-1. Inhibition of metalloproteases (MMP represents a cocktail of 38 µM GM6001 and 30 µM TAPI-1) (**a**, left panel) increased mEPSC frequency, eEPSC amplitude (**b**, left panel), and RRP size (**c**, left panel) when compared to control neurons (ctrl), while neurons exposed to NX-1βe showed the opposite effects—compared to ctrls. In NL-1 KO littermates, the inhibition of metalloproteases was without effect (**a**–**c**, right panels). All panels: top: representative traces; bottom: summarized and averaged data).

If shedding of ectodomains from endogenous proteins (including, but not limited to, NX-1β) is involved in setting the synaptic strength in neuronal cultures, then blocking metalloproteases to prevent shedding should induce compensation of the synaptic strength in the opposite direction. We therefore incubated mouse autaptic cultures (DIV 12–14 days) overnight with two broad-spectrum metalloprotease inhibitors, GM6001 (38 µM) and TAPI-1 (30 µM), which have been shown to prevent NX-1β (and NL-1) ectodomain shedding^35^. Other culture dishes from the same neuronal preparations were incubated only with NX-1βe, or left as controls. Strikingly, metalloprotease inhibitors caused an increase in mEPSC frequency, eEPSC amplitude and RRP size beyond control values (Fig. 6a–c), whereas NX-1βe again caused a decrease in those parameters (see Supplementary Fig. S8 for additional parameters from this experiment, and Supplementary Fig. S9 for similar findings in rat neurons). Immunostaining of neurons for synaptophysin and MAP2 did not show any significant differences in synaptic number or dendritic branching after overnight exposure to NX-1βe, or GM6001/TAPI-1 (Supplementary Fig. S10). These findings are consistent with the notion that regulated shedding of endogeneous ectodomains can be involved in bidirectional alterations of synaptic strength.

We next investigated whether NL-1 is a prerequisite for the physiological effects of the NX-1βe and the effect of metalloprotease inhibitors. Notably, all effects were abolished in neurons cultured from NL-1 KO mice (Fig. 6a–c). These data show that NL-1 is an obligatory intermediary key player for metalloproteases to modulate presynaptic strength, both acutely and during long-term exposure. However, it does not rule out that other metalloprotease targets—of which there are many in the synapse^41^—could also be required.

## Discussion

This study delineates roles for the NX-1β ectodomain in neuronal development (dendrite formation and neuronal survival), and in acute and chronic modulation of synaptic transmission (temporarily increasing presynaptic efficacy, while concurrently triggering down-regulation of the RRP to rebalance synaptic strength).

Neurite outgrowth is a complex process accompanying neuronal differentiation. This process involves different CAMs (e.g. NCAM, N-cadherin and L1) and neurotrophic factors (e.g. NGF and BDNF) and leads to intracellular events, including activation of receptor tyrosine kinases, Ca^2+^-influx, inhibition of actin-capping proteins and altered actin dynamics^63,64^. Our data obtained from immature neurons indicate that NX-1β and NL-1 expressed before synaptogenesis^45^ can be recruited for neuritogenesis. Indeed, it has been shown that NL-1 with the B-insert induces neurite outgrowth through interaction with NX-1^65^. Reciprocally, we show here that the soluble NX-1β ectodomain without the SS4 insert strongly induced neuritogenesis in NL-1-expressing hippocampal neurons. Interestingly, both dose–response curves are bell-shaped (present study and^50,65^), and thus the effect is not apparent at 1 nM NX-1βe (Fig. 1a) or 10 nM soluble NL-1^65^. Previous experiments did not report NX-1βe-induced neurite outgrowth^22,59^, consistent with the higher concentrations used in those studies. This result together with the fact that the employed NX-1βe is a dimer (a FC-chimera) and the finding that tetrameric and dimeric Neurexide were more effective than monomeric peptide indicates that clustering of NL-1 (or NX-1β^65^), is involved. Using Fura-imaging we show that NX-1βe induces Ca^2+^-influx via mechanisms that include N-type Ca^2+^ channels, and blocking N-type Ca^2+^-channels abolishes the neuritogenic effect of NX-1βe. Ca^2+^-influx has repeatedly been implicated in supporting growth of neurites^66–68^, and thus, clustering of NL-1 might act as a gatekeeper mechanism to allow localized Ca^2+^-influx to stimulate formation of dendrites in the part of the neuron where NL-1 is ligated. The complementary data obtained with soluble NL-1 (NX-1-dependent outgrowth of axons^65^) and NX-1βe/Neurexide [NL-1 dependent outgrowth of dendrites (Supplementary Fig. S3)] is parallel to previous findings, that NL-1 binding to NX-1β induces presynaptic specializations^69^, and that binding of NXs to NLs induces post-synaptic specializations^21^. Our data thus indicate that even before synaptogenesis, NL-1 and NX-1β can stimulate neurite outgrowth. These data were obtained in vitro and it should be stressed that in vivo there are likely many redundant pathways for synaptogenesis and neurite outgrowth, some of which are independent of NXs and NLs.

In developing cultured neurons, postsynaptic NL-1 enhanced the size of the RRP^27^, whereas cleaving of NL-1 led to a decrease in release probability (increase in PPR) within 30 min^40^. Notably, we show here that the acute application of NX-1βe or Neurexide to neurons that have already formed functional synapses increases synaptic release probability (decreases PPR) and increases mEPSC frequency within a few seconds in a NL-1 dependent manner. Two different NL-1 binding compounds (NX-1βe or Neurexide), which are produced using different protocols elicits identical results, which are abolished in NL-1 KO cells, and depends on NMDA-receptors. The two latter findings—and the known interaction between NL-1 and NMDA-receptors—make it likely that NX-1βe acts at least in part via binding to NL-1. However, it should be kept in mind that there are many other interaction partners of NX-1β (see “Introduction”), and they typically bind via sites that overlap with the NL-1 binding site. It is therefore possible that other NX binding partners are also required for the effect seen.

A generalized adaptation of both pre- and postsynaptic Ca^2+^-homeostasis seems to underlie the NX-1βe-dependent effects of synaptic release. We show that NX-1βe causes acute Ca^2+^-influx and that blocking NMDA receptors blocks the effect of NX-1βe on presynaptic release. Using presynaptic Ca^2+^-imaging, we found a small change in presynaptic Ca^2+^-homeostasis upon NX-1β application, suggesting that the mechanism could involve a direct (NL-1 dependent) effect on presynaptic boutons or involve a retrograde signal that travels from the post- to the presynapse to trigger changes in [Ca^2+^]_presynaptic_. Since the effect of neurexin ectodomain or neurexide application was acute and appeared within a few seconds, it can be effectively ruled out that Ca^2+^ or another signal could diffuse intracellularly from the postsynaptic to the presynaptic side to elicit the effect. The effects on presynaptic release could be blocked by inhibiting NMDA-receptors and might involve NMDA-receptors on either the postsynaptic or the presynaptic side^56^ of the synapse; neurexin ectodomain might interact with either or both upon binding to NL-1 in the synapse. Interestingly, it has been found that dopamine acting on D1-receptors can stimulate Ca^2+^-influx through NMDA-receptors in D2R-neurons in a reaction that requires metalloproteinase activity in an intermediate step^70^. Furthermore, NX-1β has been shown to regulate presynaptic calcium channels via a retrograde synaptic endocannabinoid signaling pathway^32^. NX-1βe does not contain the C-terminal end of Neurexin, and Neurexide, which only contains the minimal NL-1 binding sequence, elicited a similar effect. Thus, the cytoplasmic tail of neurexin is not required for NX:NL-dependent strengthening of the presynapse, just as it is dispensable for synapse formation^71^. It is in principle possible that application of NX-1βe could break up existing NX:NL-dimers, leading to relaxation of a persistent presynaptic inhibition by the cytoplasmic neurexin-tail. Alternatively, or in addition, the acute increase in postsynaptic [Ca^2+^]_i_ upon NX-1βe application might represent a further augmentation of a signaling process already going on tonically^32,55,72,73^.

Whereas short-term exposure to NX-1βe increases the release probability, longer-term treatment results in a decrease in the RRP, which is complete within 2 h. A significant effect on mEPSC amplitude was seen in one experiment (Supplementary Fig. S8), but not in others (Supplementary Fig. S7, Fig. 5b). This indicates that the effects were predominantly presynaptic. The fact that the down-regulation is abolished in the NL-1 KO, by blocking ionotropic glutamatergic transmission, or by inhibiting activity with TTX, suggests that it is a downstream consequence of the initial synaptic strengthening induced by NX-1βe, rather than a separate phenomenon. The activity dependent reduction in RRP size is the hallmark of presynaptic homeostatic mechanisms^61,74,75^, which counteracts the short-term NX-1βe-induced increase in release probability. Previously, a role for α-NXs in synaptic homeostasis at the mouse neuromuscular junction has been described^76^. Our data imply the acute effects of NX-1βe on synaptic function can be similarly controlled using homeostatic mechanisms in central synapses.

This mechanism might explain the reduction in RRP size upon K^+^-depolarization in autaptic hippocampal neurons^77^, which indeed is also expected to lead to increased Ca^2+^ influx, and activity-induced enhancement of metalloprotease activity^35,40,78^. Either K^+^ or NX-1βe application will increase synaptic transmission and activity, which might stimulate metalloproteases to cleave NL-1^35^. This suggestion aligns with data indicating presynaptic down-regulation upon acute cleavage of NL-1^40^ and also with the increase in eEPSC size and presynaptic vesicle pool seen upon NL-1 overexpression^26,27,30^. Consistent with this model we show that overnight incubation with a mixture of two metalloprotease inhibitors induces an increase in RRP and eEPSCs size. Metalloproteases are likely to cleave many different synaptic proteins; however, strikingly we found that they did not modulate synaptic function in NL-1 KO neurons, indicating that NL-1 is a necessary component—not necessarily the only one—for their action in cultured neurons.

Collectively, our findings provide evidence for the involvement of the NX-1β ectodomain in neurite formation and in acute and chronic regulation of synaptic transmission. Using a synthetic peptide, modeled based on the minimal NL-1 binding sequence, Neurexide, we show that this effect can be mimicked by pharmacological manipulation. This adds to mounting evidence that NXs are intricately involved in synaptic plasticity mechanisms.

## Methods

### Animal experiments

Permission to keep and breed knockout mice for this study was obtained from The Danish Animal Experiments Inspectorate. Neuroligin-1 KO mice^25^ were kindly provided by Dr. Nils Brose (Max-Planck-Institute for Experimental Biology, Göttingen), and maintained in the heterozygous condition. Heterozygous crosses were used to recover knockout animals (NL-1 KO). All animals used for experiments were genotyped using a PCR-protocol^79^. All animals were maintained in an AAALAC-accredited stable and all protocols were performed in accordance with institutional guidelines as overseen and approved by the Institutional Animal Care and Use Committee (IACUC) of the University of Copenhagen. Adult mice were sacrificed by cervical dislocation; embryos were sacrificed by decapitation.

### Peptides and proteins

The Neurexide peptide (ARPSTRADRA) was synthesized as a monomer, a dimer or a tetramer coupled to a lysine backbone. Scrambled (RDATAPRSAR, DARRSATARP), reversed (ARDARTSPRA), and alanine-substituted derivatives of Neurexide were synthesized as tetramers (> 80% purity; Schafer-N, Copenhagen, Denmark). Human neurexin-1β Fc-chimera (NX-1βe) contained two ectodomains and was obtained from R&D Systems (Cat#:5268-NX, Minneapolis, MN, USA).

### Primary hippocampal and cerebellar granule neuron cultures for neurite outgrowth and neuronal survival

Hippocampal neurons were isolated from Wistar rats on embryonic day 19 or C57Bl6 mice on embryonic day 18 (Charles River, Sulzfeld, Germany) essentially as previously described^64,80^. Cerebellar granule neurons were isolated from Wistar rats on postnatal day 7, as described^80^. In short, dissociated cultures of neurons were seeded on LabTek Permanox slides (Nunc, Roskilde, Denmark) at a density of 12,500 cells/cm^2^ on top of a confluent monolayer of mouse fibroblastoid L929 cells to improve the survival of electroporated cells (NL-1 knockdown cultures; DIV1), or directly on poly-l-lysine (wildtype cultures used for neurite outgrowth analysis; DIV1 and wildtype cultures used for morphological analysis; and DIV7 cultures) as previously described^81^. The cultures were incubated in neurobasal medium supplemented with 2% (v/v) B27, 0.4% (w/v) BSA, 2 mM GlutaMAX, 20 mM HEPES, 100 U/ml penicillin, and 100 μg/ml streptomycin (all purchased from Gibco BRL, Paisly, UK) at 37 °C and 5% CO_2_. Immediately after seeding, soluble NX-1β or Neurexide (see below) was added to the cultures. For knockdown of Neuroligin-1 (NL-1) expression, the neurons were transfected with a p-GFP-V-RS vector that encodes short-hairpin RNA targeting NL-1 (OriGene, Rockville, MD, USA) using a nucleofector device and a Rat Neuron Nucleofector kit (Amaxa, Gaithersburg, MD, USA), and seeded in neurobasal A medium supplemented with 5% (v/v) horse serum, 2% (v/v) B27, 2 mM GlutaMAX, 100 μg/ml streptomycin, 100 U/ml penicillin and 2.5 μg/ml fungizone for 24 h at 37 °C in 5% CO_2_. As a control, neurons were transfected with a pGFP-V-RS vector that encodes scrambled short-hairpin RNA. Only cells positive for GFP fluorescence were analyzed. Pharmacological inhibitors were added to the cultures 10 min prior to the addition of 42 pM soluble NX-1β or 17 µM Neurexide. ω-Conotoxin MVIIA (Nordic Biosite, Täby, Sweden) was used to inhibit N-type voltage-dependent calcium channels. Ryanodine and Xestospongin C (both from Merck) were used to inhibit intracellular Ca^2+^ release.

### Analysis of neuronal survival

#### Potassium deprivation

Cerebellar granule neurons were seeded at a density of 62,500 cells/cm^2^ in eight-well poly-d-lysine (0.01 μg/cm^2^)-coated LabTek Permanox slides (Nunc) and grown for 7 days at 37 °C and 5% CO_2_^82^. Apoptosis was induced by reducing the potassium levels in the medium from 40 to 5 mM and increasing concentrations of NX-1βe or Neurexide were added. Forty-eight hours later, the cells were fixated, stained and numbers of survived neurons was determined as previously described^82^.

#### Oxidative stress

Hippocampal neurons were grown as described for the potassium deprivation model. On DIV 7, NX-1βe or Neurexide were added. One hour later, H_2_O_2_ (Sigma-Aldrich) was added to a final concentration of 60 µM, and the cultures were incubated for 24 h, fixated, stained, and analyzed similarly to the potassium deprivation model.

### Analysis of neurite outgrowth

The DIV1 neuronal cultures (Figs. 1a–c, 2c–f and Supplementary Fig. S1a–c, S2b–f) were fixed in PBS with 3.7% formaldehyde and immunostained with rabbit anti-growth-associated protein (GAP)-43 antibody (Millipore, Bioscience Research Reagents, Denmark), visualized with Alexa-conjugated goat anti-rabbit (Invitrogen, Denmark), and micrographs were recorded using a systematic random mode and evaluated as previously described^64,83^.

The DIV7 cultures (Supplementary Fig. S3) were fixed and immunostained with either mouse anti-MAP2 antibody diluted 1:400 (BD Pharmingen, CA, USA) or mouse anti-SMI312 antibody diluted 1:1000 (Covance, Princeton, NJ, USA), visualized with Alexa-conjugated goat anti-mouse (Invitrogen), and mounted with antifade mounting medium (Dako, Glostrup, Denmark). Micrographs were recorded and evaluated as for the DIV1 cultures.

### Surface plasmon resonance analysis

The analysis was performed with a Biacore 2000 machine (GE Healthcare, Hilleroed, Denmark). NL-1 (cat#4340-NL; R&D Systems) was immobilized on a CM4 sensor chip using an amine coupling kit (GE Healthcare). Immobilization was performed at 5 µl/min, and the activation and deactivation time was 7 min. Injections of 45 μl NL-1 (0.033 μg/μl) in 10 mM sodium-acetate, pH 4.0, resulted in immobilization of ~ 2800 resonance units (RU). The analysis was performed at 25 °C using Ca^2+^-supplemented HBS-P (10 mM HEPES (pH 7.4), 150 mM NaCl, 0.005% (v/v) Surfactant P20, 3 mM CaCl_2_) as running buffer for analysis of NX-1β or Neurexide binding to NL-1. The NX-1β Fc chimera (0.05–0.8 μM) or Neurexide (1.25–20 µM) was diluted in running buffer, and injected at a flow rate of 30 μl/min. Regeneration was performed with an injection of 15 μl of 1 M NaCl_2_. The data were analyzed by non-linear curve fitting using the software package BIAevaluation v.4 (GE Healthcare). The curves were fitted to a 1:1 Langmuir binding model, and rate and equilibrium constants were calculated.

### Primary hippocampal cultures for electrophysiology

Hippocampi were dissected from embryonic day 19 Wistar rat (Charles River) or embryonic day 18 *neuroligin-1* null mutant (−/−) mice and control littermates (+/+). Hippocampal neurons were plated at 2500/cm^2^ on micro islands of mouse (NMRI) glia, as described^84^. Glial islands were obtained by first coating glass coverslips with 0.15% agarose. After drying and UV sterilization custom-made rubber stamps were used to print dots (islands) using a substrate mixture containing 0.25 mg/ml rat tail collagen and 0.4 mg/ml poly-d-lysine dissolved in 17 mM acetic acid; glial cells were plated at 4800/cm^2^ two days before use, ensuring confluent coverage of microdot islands. Soluble NX-1β (R&D Systems) was applied at a concentration of 5 ng/ml in long-term (> 10 days, added on DIV1), short-term (~ 2 h) and acute application experiments. Neurexide tetramer was applied at an end-concentration of 47.6 µg/ml. Matrix metalloprotease mixture was added 16 h before recording and contained GM6001 (38 µM, Millipore) and TAPI-1 (30 µM, Calbiochem).

### Neuronal morphology

At DIV9 autaptic hippocampal neurons were treated with soluble NX-1βe, or the matrix metalloprotease mixture (see above), and fixed at DIV10 for 15 min in PBS with 4% paraformaldehyde. Cells were permeabilized for 10 min with 0.02% Tween-20 in PBS (PBST), and incubated for 1 h with blocking solution (4% normal goat serum in PBST). The fixed neurons were incubated overnight at 4 °C with monoclonal anti-Synaptophysin-1 (Synaptic Systems, cat no. 101011, 1:750), and chicken polyclonal anti-MAP2 (Abcam, cat no. ab5392, 1:1500) antibodies. After washing, cells were incubated for 1.5 h with Alexa-labeled secondary antibodies [Molecular Probes, goat anti-chicken (cat no. A-11039, 1:1000), goat anti-mouse (cat no. A-21235, 1:1000)], and mounted on glass slides with FluorSave. Confocal images of autaptic neurons were recorded using a Zeiss LSM 710 confocal laser point scanning system (Zeiss, 20×/0.8 air objective, 488 nm laser Argon 25 mW, 633 nm laser NeHe 5 mW). A (semi)-automated analysis routine in MATLAB (Synapse and neurite detection, SynD) was used to examine dendritic arborization and synapse density/localization^85^.

### Electrophysiological recordings

Isolated neurons cultured from embryonic Wistar rats, NL-1 KO mice and their wildtype littermates were recorded on DIV10-14. The patch pipette solution contained (in mM): 136 KCl, 18 HEPES, 4 Na-ATP, 4.6 MgCl_2_, 4 K_2_-ATP, 15 Creatine Phosphate, 1 EGTA and 50 U/ml Phosphocreatine Kinase (300 mOsm, pH 7.30). The standard external medium used contained 2 mM/2 mM Ca^2+^/Mg^2+^ [in mM: 140 NaCl, 2.4 KCl, 2 CaCl_2_, 2 MgCl_2_, 10 HEPES, 14 Glucose (300 mOsm, pH 7.30)]. Cells were whole-cell voltage clamped at − 70 mV with a double EPC-10 amplifier (HEKA Elektronik, Lambrecht/Pfalz, Germany) under control of Patchmaster v2x32 software (HEKA Elektronik). Currents were low-pass filtered at 3 kHz and stored at 20 kHz. Patch pipettes were pulled from borosilicate glass using a multi-step puller (P-897; Sutter Instruments). Pipette resistance ranged from 3 to 5 MΩ and was compensated to 85%. Only cells with series resistances < 15 MΩ were included in analysis. All recordings were made at room temperature. EPSCs were evoked by depolarizing the cell from − 70 to 0 mV for 2 ms. A fast local multi-barrel perfusion system (Warner SF-77B, Warner Instruments) was used to establish acute application of NX-1βe or Neurexide. For dendritic Ca^2+^-measurements in mature neurons Fura-2 (200 µM) was added to the internal medium and infused for ~ 20 min to allow optimal infusion. Neurons used for presynaptic Ca^2+^-measurements were transduced with syGCaMP2^58^ expressing lentiviral particles on DIV1 and recorded on DIV 10–14. Experiments were conducted in regular external recording medium on an inverted Zeiss Axiovert 200 microscope equipped with an F-Fluar 40×/1,30 numerical aperture oil-immersion objective (Carl Zeiss Microscopy). Fluorophores were excited by a monochromator (Polychrome V, TILL Photonics) controlled by TILLVision, and images (1376 × 1040 pixels) were acquired with a cooled digital 12-bit CCD camera (SensiCam, PCO-Tech). A custom analysis procedure in Igor Pro (Wavemetrics Inc.) was used for offline analysis of evoked and sucrose responses. Spontaneous events were detected using Mini Analysis program (Synaptosoft). Fiji (ImageJ) was used for analysis of fluorescence data.

### Ca^2+^-measurements in immature rat neurons

Rat neurons were prepared as described above (primary hippocampal and cerebellar granule neuron cultures for neurite outgrowth and neuronal survival). After 24 h the neuronal cultures were treated with 6 µM Fura-2AM (Sigma) for 30 min at 37 °C and 5% CO_2_ (dark). After incubation with Fura-2AM the cells were washed 2 times with prewarmed supplemented neurobasal medium. After washing the treated cells were put back into the incubator for 10 min at 37 °C and 5% CO_2_ (dark) to complete enzymatic removal of the acetoxymethyl (AM) group of internalized Fura-2AM. Imaging experiments were conducted on the experimental setup described above, but without patching the cells. Bath applications were performed using a local gravity-driven perfusion system.

### Statistics and graphical presentation

For neuronal outgrowth data, *n* denotes the number of cultures; a minimum of 150 neurons per condition were included. For electrophysiological recordings, the results are shown as average ± SEM with *n* referring to the number of cells for each group unless otherwise stated. When comparing two groups, the variances were first compared using an *F* test. In case of homoscedastic data (*F* test insignificant), we tested differences between group means using a Student's *t* test. In case of heteroscedastic data (*F* test significant), we tested difference between group medians using a Mann–Whitney *U* test. Significance was assumed when p < 0.05. Graphical presentation and statistical testing was performed using SigmaPlot 12.3 (Systat Software Inc). In figures, the significance levels are indicated by asterisks (*p < 0.05; **p < 0.01; ***p < 0.001).

For neurite outgrowth and neuronal survival, the statistical analyses and graphical presentations were performed using Prism software (GraphPad, San Diego, CA, USA). Differences between groups were analyzed using two-tailed Student’s *t* test or one-way repeated-measures analysis of variance (ANOVA) followed by Dunnett’s post hoc test. The data are presented as mean ± SEM. Significance differences from designated controls are indicated by asterisks (*p < 0.05, **p < 0.01, ***p < 0.001).

## Supplementary information

Supplementary Figures.
