# Supplementary material for: The soluble neurexin-1β ectodomain causes calcium influx and augments dendritic outgrowth and synaptic transmission
Source: Sci Rep. 2020 Oct 22;10:18041. doi: 10.1038/s41598-020-75047-z (PMC7582164; doi:10.1038/s41598-020-75047-z)
Supplement: Supplementary file 1 — Supplementary Figures. [file 41598_2020_75047_MOESM1_ESM.pdf]

Supplementary Information

for

The soluble neurexin-1β ectodomain causes calcium influx and augments dendritic outgrowth and synaptic transmission

Keimpe D. B. Wierda[1,3*], Trine L. Toft-Bertelsen[1,2,3,*], Casper R. Gøtzsche[2], Ellis Pedersen[1], Irina Korshunova[2], Janne Nielsen[2], Marie Louise Bang[2], Andreas B. Kønig[2], Sylwia Owczarek[2], Michelle D. Gjørlund[2], Melanie Schupp[1], Elisabeth Bock[2], and Jakob B. Sørensen[1*]

[1]*Neurosecretion Group, Department of Neuroscience, University of Copenhagen, Blegdamsvej 3B, DK-2200 Copenhagen N, Denmark.*

[2]*Laboratory of Neural Plasticity, Department of Neuroscience, University of Copenhagen, Blegdamsvej 3, DK-2200 Copenhagen N, Denmark*

[3] Contributed equally to this work

*Correspondence should be addressed to J.B.S. (jakobbs@sund.ku.dk), K.D.B.W (keimpe.wierda@kuleuven.vib.be) or T.L.T-B. (trineto@sund.ku.dk).

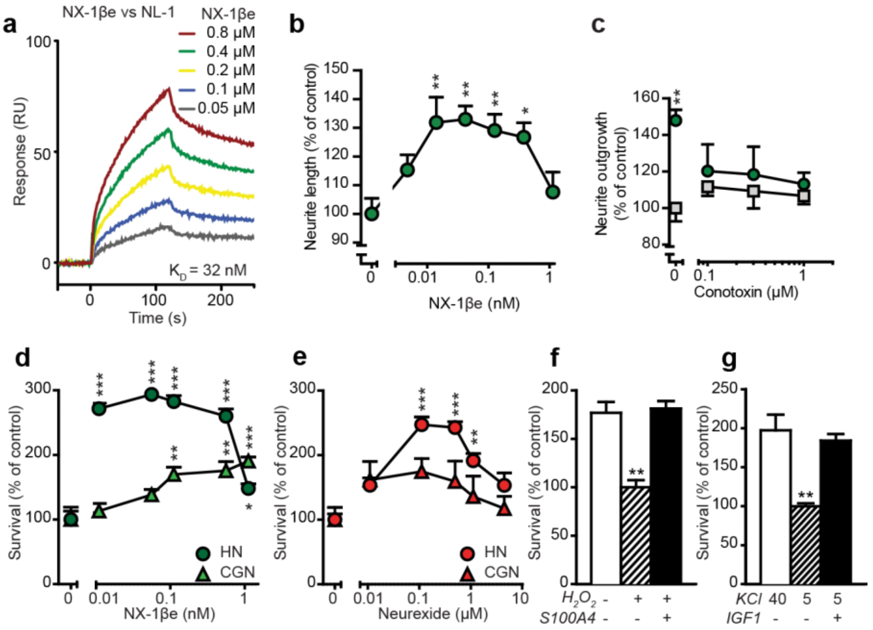

Supplementary Figure S1. NX-1β ectodomain stimulates neurite outgrowth and neuronal survival. (a) Binding of NX-1βe to NL-1 immobilized on a CM4 sensor chip. NX-1βe was injected at increasing concentrations over the chip at a flow rate of 30 μl/min ($n = 3$). The results are presented as sensorgrams. RU, resonance units. **(**b) NX-1βe promoted neurite outgrowth in mouse hippocampal neurons (E18). (c) ω-conotoxin MVIIA - an inhibitor of *N*-type VDCCs - inhibited the effect of NX-1βe on neurite outgrowth. (d) NX-1βe and (e) Neurexide both increased survival of hippocampal neurons (HN) in the oxidative stress model and on cerebellar granule neurons (CGN) in the potassium deprivation model. (f) Addition of 15 μM S100A4 (positive control) counteracted the cell death in HN after oxidative stress induced by H_2O_2. (g) Addition of 6.6 nM insulin-like growth factor 1 (IGF1; positive control) counteracted cell death in CGN induced by changing the potassium levels in the medium from 40 mM to 5 mM.

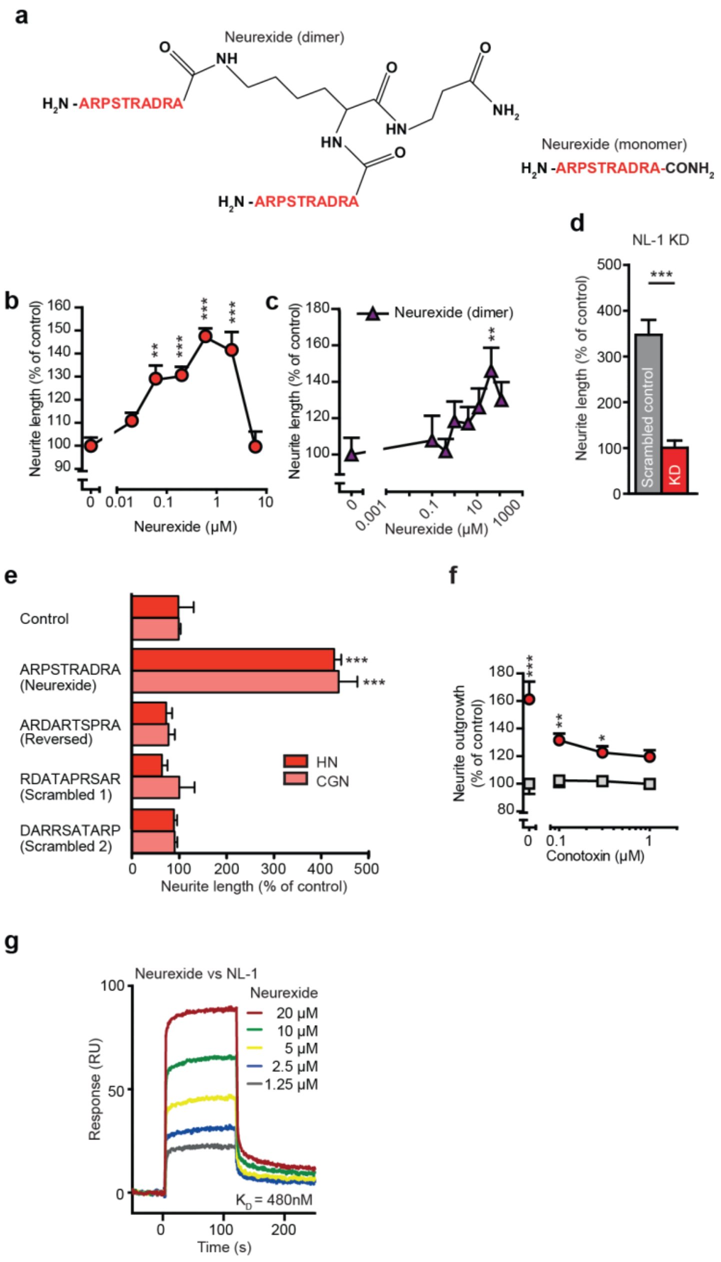

Supplementary Figure S2. Neurexide promotes survival and affect calcium-mediated signaling mechanisms involved in neurite outgrowth. (a) Structure of monomeric and dimeric Neurexide. (b-f): DIV1, n = 4 cultures. (b) Neurexide promoted neurite outgrowth in mouse hippocampal neurons (E18). (c) Neurexide produced as a dimer also increased neurite outgrowth, but only at higher concentrations (rat neuron cultures). (d) Knockdown of NL-1 in rat neuronal cultures abrogated the effect of Neurexide. (e) Tetrameric neurexide (17 µM) with reversed or scrambled amino acid residue order did not induce neurite outgrowth. (f) ω-conotoxin MVIIA inhibited the neuritogenic effects of Neurexide. (g) Binding of Neurexide to NL-1 immobilized on a CM4 sensor chip. Neurexide was injected at increasing concentrations over the chip at a flow rate of 30 µl/min (n = 2). *: $p<0.05$, **: $p<0.01$, ***: $p<0.001$, compared to untreated control (repeated-measures one-way ANOVA, follow by Dunnett’s post hoc test, *n* = 3-5). Data are shown as mean with S.E.M.

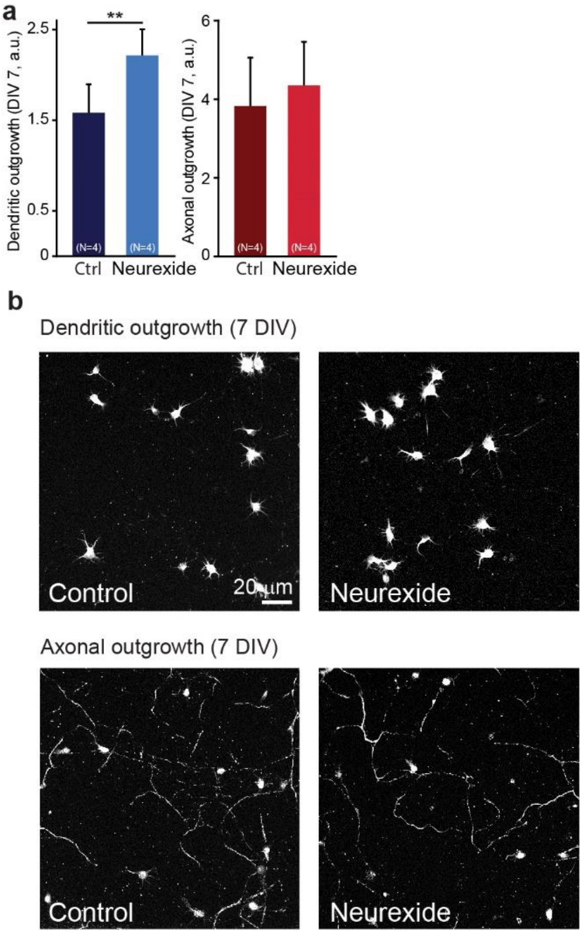

Supplementary Figure S3. Neurexide stimulates dendritic, not axonal, outgrowth in immature neurons (DIV 7).

(a) Quantification of dendritic and axonal outgrowth using immunostaining against MAP2 (dendritic marker) and Neurofilament (axonal marker), DIV7, n = 4 cultures. The neurites stimulated by Neurexide application were dendrites. ** $p<0.01$ (paired *t*-test). (b) Example immunofluorescence images stained for MAP-2 (dendritic marker) or SMI312 (axonal marker) and treated with Neurexide (right), or left untreated as control (left).

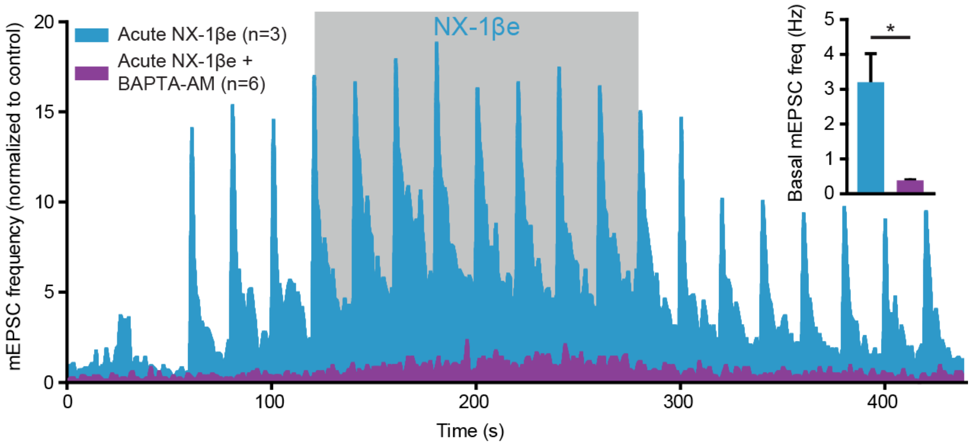

Supplementary Figure S4. The mEPSCs stimulated by the NX-1βe depend on intracellular calcium. Incubation with BAPTA-AM (a cell permeant calcium chelator) almost abolished spontaneous release, both in the absence and presence of NX-1βe. Control cells were treated with NX-1βe according to the normal protocol.

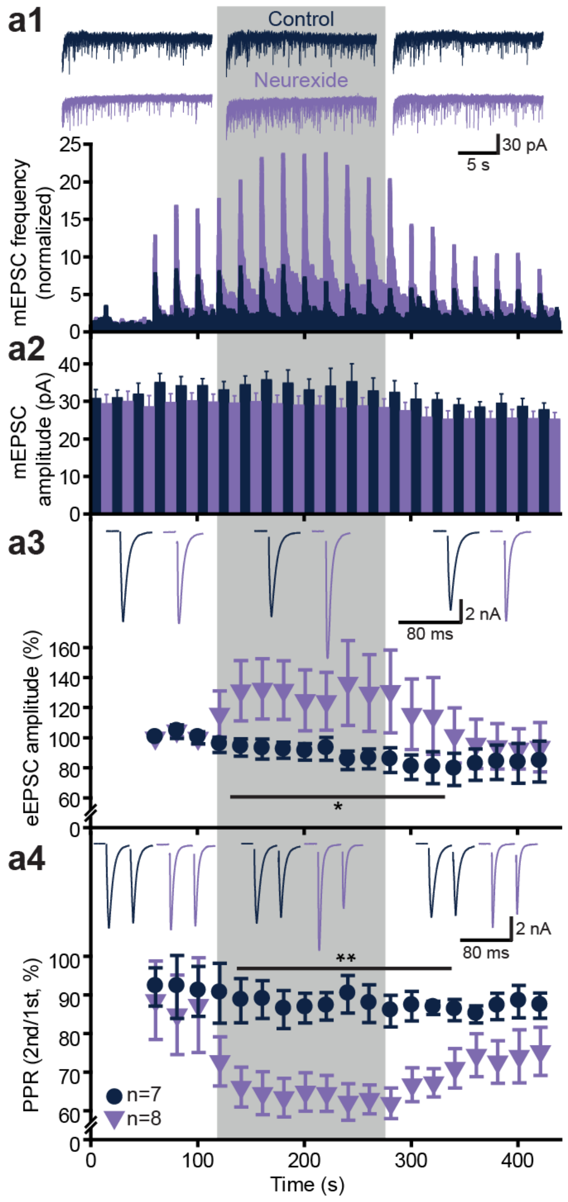

Supplementary Figure S5. Acute exposure to Neurexide stimulates glutamatergic synaptic transmission. Autaptic glutamatergic neurons were patch-clamped in whole cell voltage-clamp configuration and stimulated by action potential pairs (inter stimulus interval 50 ms) every 20 s. (a1) mEPSC frequency was quantified between stimuli, together with mEPSC amplitude (a2) eEPSC amplitude (a3) and Paired-Pulse Ratio (PPR) (a4) for each paired stimulation. Neurexide (10 µM) was applied in the bath (shaded area). This reversibly increased the mEPSC frequency, and the eEPSC amplitude, whereas the PPR was reduced, indicating increased release probability. Note the post-stimulation induced (Ca^{2+}-dependent) periodic bursts in mEPSC release frequency.

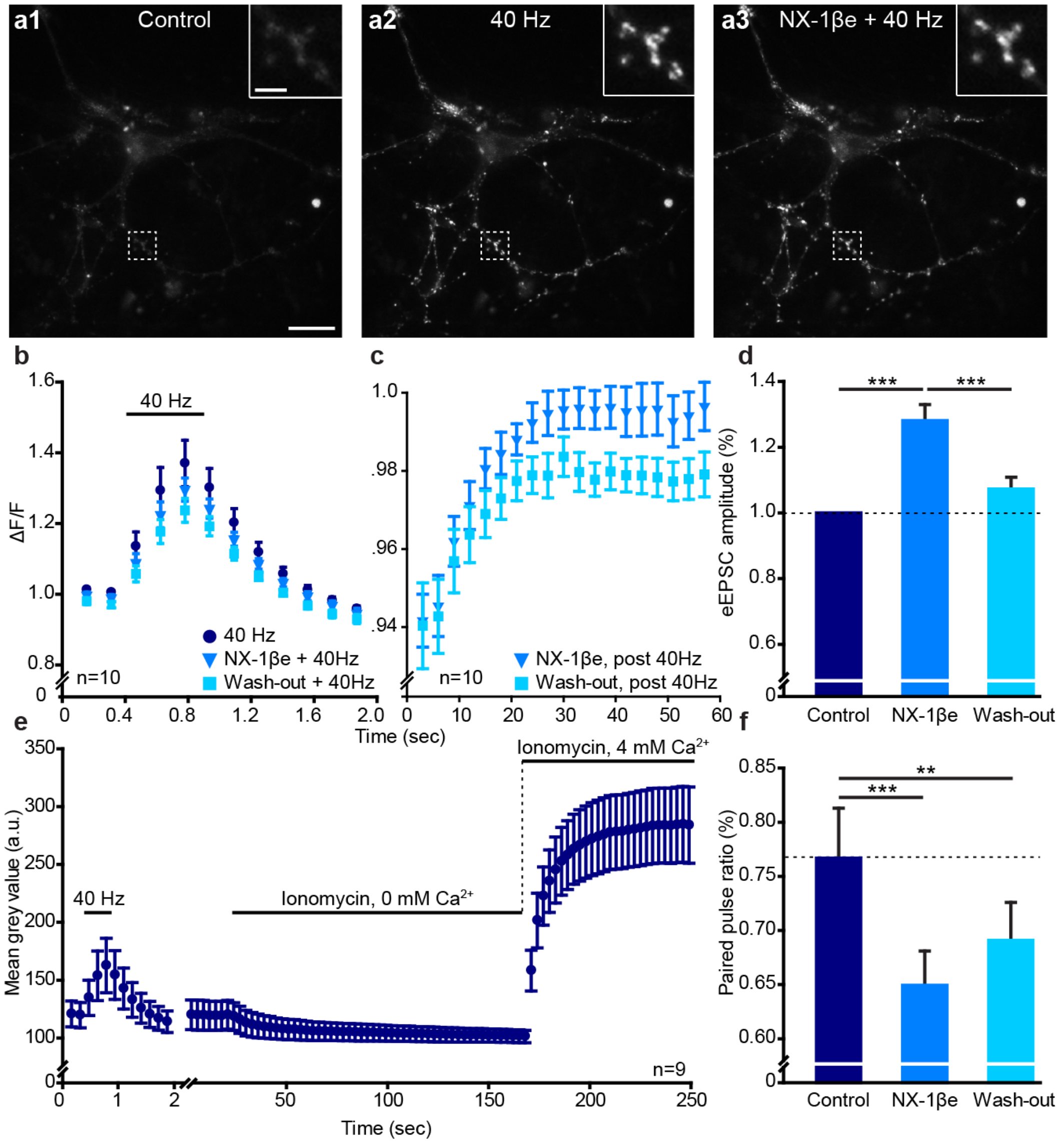

Supplementary Figure S6. The NX-1β ectodomain increases basal presynaptic [Ca^{2+}] but not calcium influx during train stimulations.

(a1-a3) Fluorescence images of neuron expressing the fluorescent Ca^{2+}-indicator syGCaMP2 before stimulation (a1), during a 40 Hz train (a2), and during a 40 Hz train in the presence of NX-1βe (a3). Stimulation led to Ca^{2+}-influx and activated the fluorescence of syGCaMP2. (b) Relative fluorescence increase (ΔF/F) during a 40 Hz-train in the absence and the presence of NX-1βe, and after wash-out.. (c) Relative fluorescence (ΔF/F) after 40 Hz-train stimulations in the presence of NX-1βe and after wash-out. Note that the calcium concentration undershoots following the train, presumably due to activity dependent calcium clearance, and then relaxes back up to baseline. NX-1βe mildly increased basal calcium levels 1 minute after the 40 Hz train. (d) Evoked EPSC amplitude was augmented by NX-1βe application. (e) 40 Hz train followed by ionomycin treatment in the absence and presence of calcium. These data show that the fluorescence of syGCaMP2 was not saturated by the used 40 Hz trains. (f) Paired pulse ratio (PPR) was decrease by NX-1βe application.

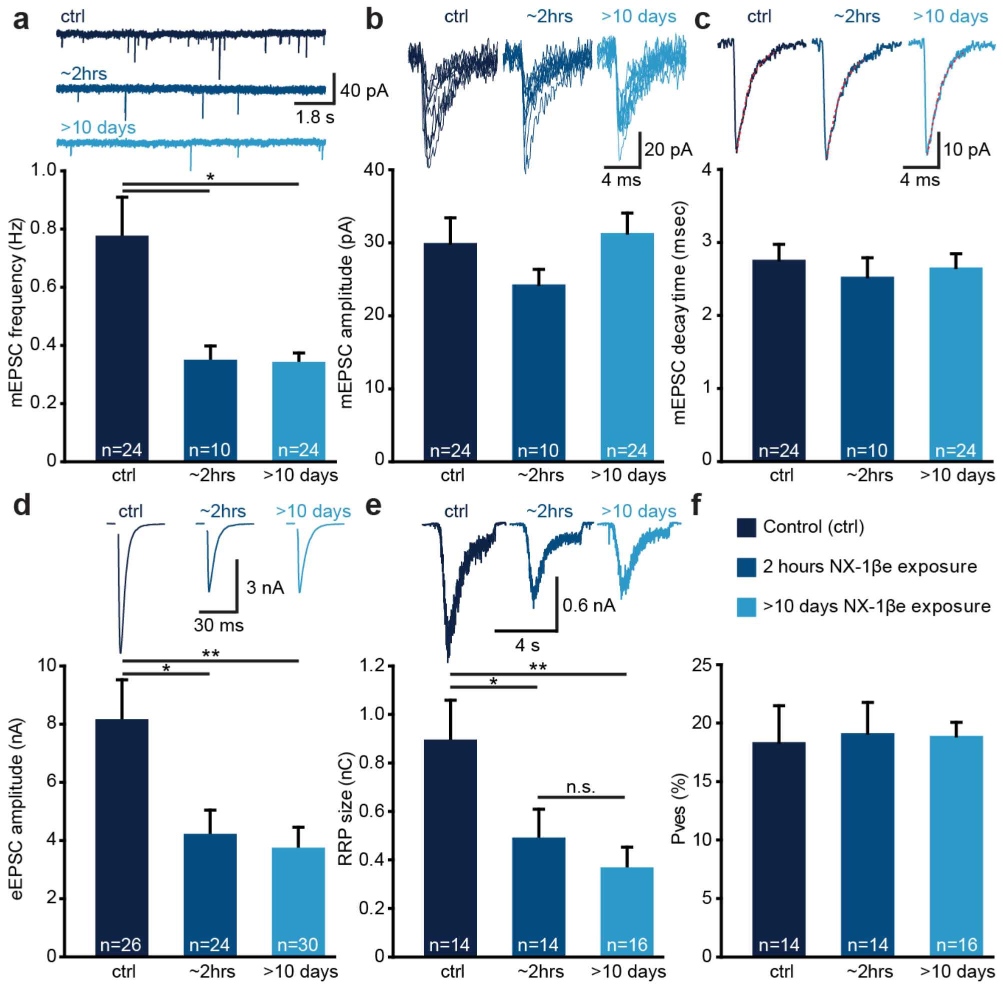

Supplementary Figure S7. Down-regulation of Readily Releasable Pool size by exposure to soluble NX-1β ectodomain within 2 hours.

Exposure to NX-1βe for either >10 days or for ~2 hours led to a significant decrease in mEPSC frequency (a), eEPSC amplitude (d), and RRP size (e) as probed by 500 mOsm sucrose application. In contrast, the NX-1βe did not affect mEPSC amplitude (b), mEPSC decay time (c) or the vesicular release probability (f). The latter quantity is calculated by dividing the charge of a single EPSC by the initial charge of the RRP.

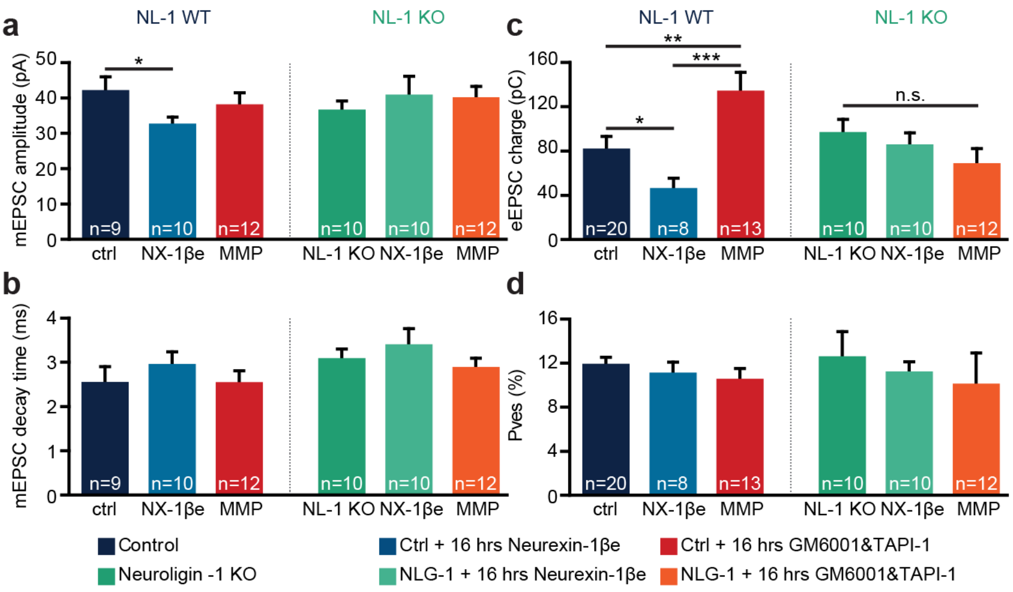

Supplementary Figure S8. NL-1 is essential for NX-1βe/matrix metalloproteases-induced modulation of synaptic transmission. (a-b) mEPSC amplitude was slightly depressed (a), while decay time (b) was unaffected by long-term NX-1βe treatment. MMP blocker treatment did not change mEPSC amplitude (a) or decay time (b). (c) NX-1βe and MMP blocker treatment respectively decreased and increased eEPSC charge (c, left), which depended critically on the presence of NL-1 (c, right). (d) Vesicular release probability was not affected by NX-1βe or MMP blockers and was comparable between NL-1 WT and KO neurons (d). *: $p<0.05$, **: $p<0.01$, ***: $p<0.001$ (Student's t-test, two-tailed, compared to control). n.s.= non-significant.

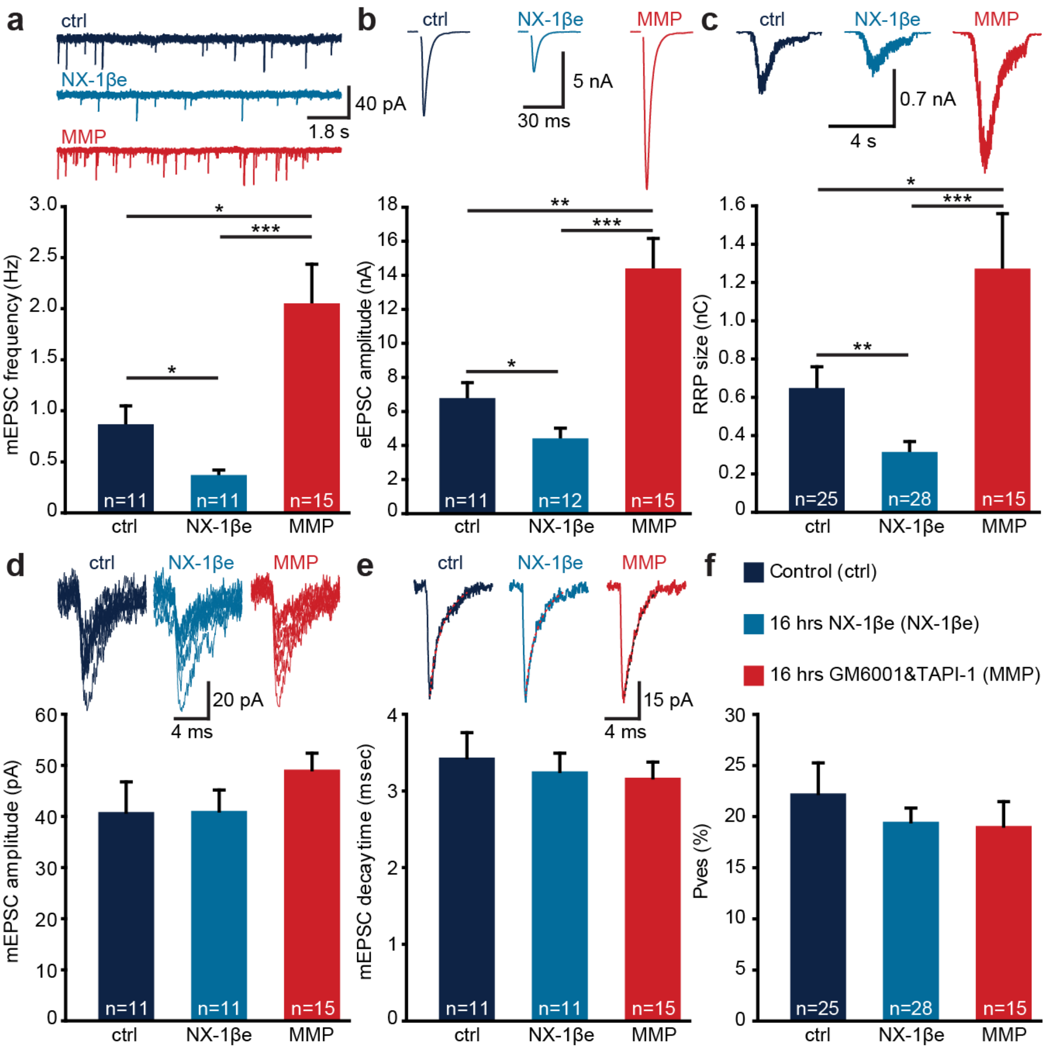

Supplementary Figure S9. Shedding of endogenous proteins by metalloproteases affects synaptic transmission in rat neuronal cultures. Inhibition of metalloproteases (MMP represents a cocktail consisting of 38 µM GM6001 and 30 µM TAPI-1) increased mEPSC frequency (a, top: representative traces. Bottom: average data), eEPSC amplitude (b), and RRP size (c) when compared to control neurons, while neurons exposed to NX-1βe again showed the opposite effects. None of the conditions affected mEPSC amplitude (d), mEPSC decay time (e) or the vesicular release probability (f).

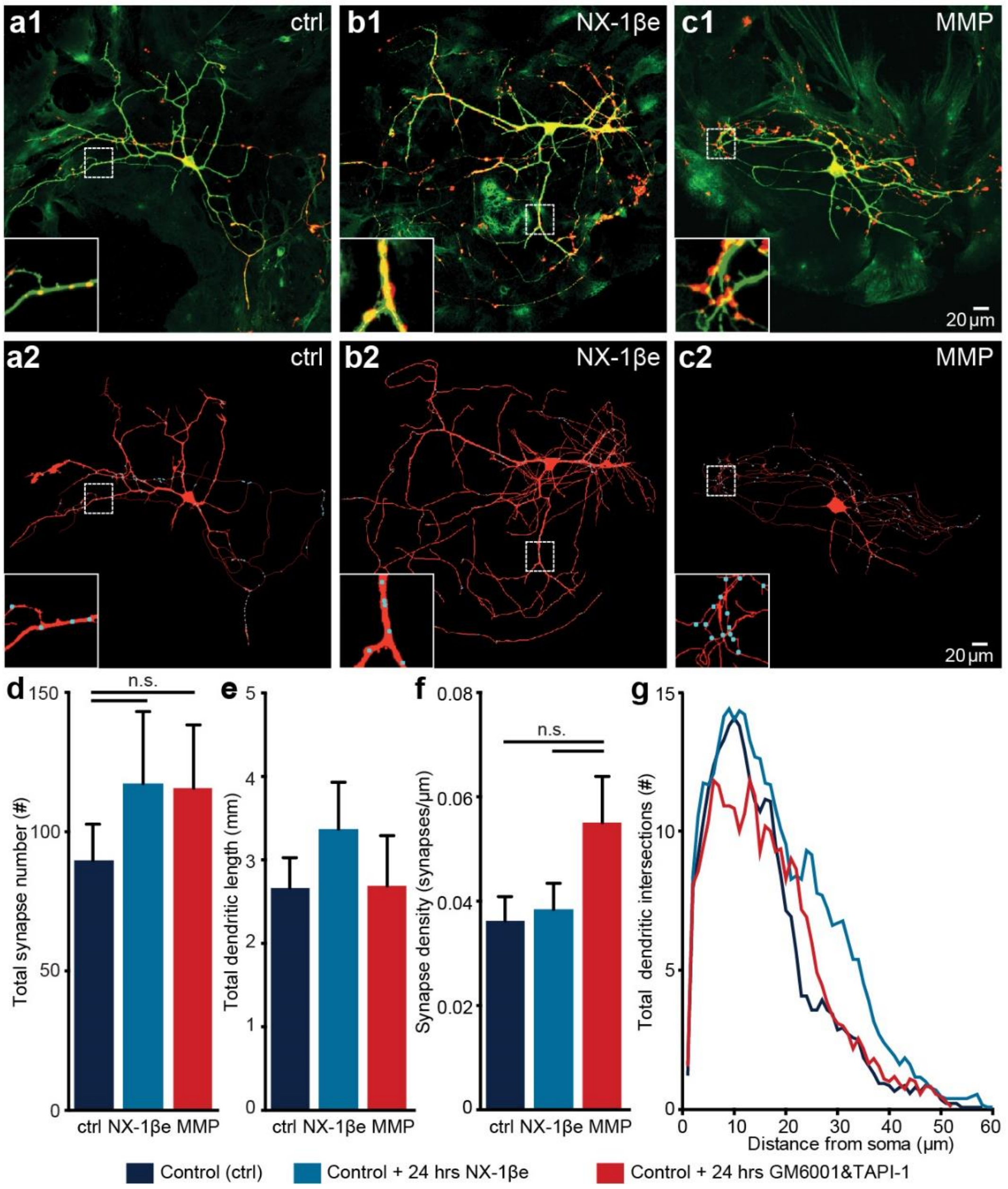

Supplementary Figure S10. Overnight exposure to NX-1βe or metalloproteases does not affect dendritic organization or synapse number in developed isolated hippocampal rat neurons. Example of autaptic hippocampal control neuron (a1) and neuron treated overnight with NX-1βe (b1) (green: MAP2, red: Synaptophysin-1) or a cocktail of metalloprotease blockers (c1). (a2-c2) Semi-automated dendrite tracing and synapse detection program was used to analyze neuronal morphology, dendrite length, synapse number and synapse density (Schmitz et al., 2011). Pretreatment with NX-1βe or MMP blockers did not significantly affect total synapse number (d), total dendrite length (e), synapse density (f) or dendritic organization (g) of isolated autaptic hippocampal rat neurons (DIV10; Student's t-test, two-tailed).
